# Crystal structure of tri­phenyl(vinyl)­phospho­nium tetra­phenyl­borate

**DOI:** 10.1107/S1600536814021357

**Published:** 2014-09-30

**Authors:** Joseph L. Bradfield, Richard A. Braun, Frankie White, Jeffrey M. Hendrich, Richard E. Sykora, Arsalan Mirjafari

**Affiliations:** a Department of Chemistry and Physics, Florida Gulf Coast University, Fort Myers, FL 33965, USA; b University of South Alabama, Department of Chemistry, Mobile, AL 36688, USA

**Keywords:** crystal structure, ionic salt, vinyl­tri­phenyl­phospho­nium, tetra­phenyl­borate

## Abstract

The title ionic salt, C_21_H_20_P^+^·C_24_H_20_B^−^, crystallized with two independent vinyl­tri­phenyl­phospho­nium cations and two independent tetra­phenyl­borate anions per asymmetric unit. These four independent moieties contain nearly perfect tetra­hedral symmetry about their respective central C atoms. In the crystal, there are no π-stacking or other inter­molecular inter­actions present.

## Related literature   

For background to the study of phosphine compounds, see: Bellina *et al.* (2012 ▶). For information on ionic liquids, see: Chowdhury *et al.* (2007 ▶).
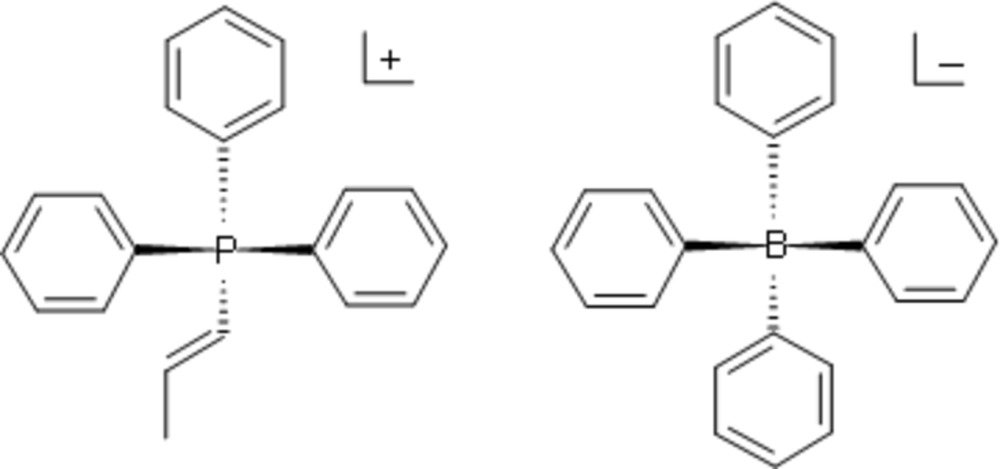


## Experimental   

### Crystal data  

C_21_H_20_P^+^·C_24_H_20_B^−^
*M* *_r_* = 622.55
Monoclinic, 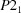

*a* = 9.2752 (4) Å
*b* = 35.7838 (15) Å
*c* = 10.9515 (5) Å
β = 100.721 (4)°
*V* = 3571.4 (3) Å^3^
*Z* = 4
Mo *K*α radiation
μ = 0.11 mm^−1^
*T* = 180 K
0.43 × 0.18 × 0.17 mm

### Data collection  

Agilent Xcalibur, Eos diffractometer
Absorption correction: multi-scan (*CrysAlis PRO*; Agilent, 2014 ▶) *T*_min_ = 0.958, *T*_max_ = 1.000
24267 measured reflections
12797 independent reflections
9444 reflections with *I* > 2σ(*I*)
*R*_int_ = 0.038

### Refinement  

*R*[*F* ^2^ > 2σ(*F* ^2^)] = 0.058
*wR*(*F* ^2^) = 0.105
*S* = 1.01
12797 reflections
849 parameters
1 restraint
H-atom parameters constrained
Δρ_max_ = 0.35 e Å^−3^
Δρ_min_ = −0.23 e Å^−3^
Absolute structure: Flack (1983 ▶)
Absolute structure parameter: 0.02 (7)

### 

Data collection: *CrysAlis PRO* (Agilent, 2014 ▶); cell refinement: *CrysAlis PRO*; data reduction: *CrysAlis PRO*; program(s) used to solve structure: *SHELXS97* (Sheldrick, 2008 ▶); program(s) used to refine structure: *SHELXL97* (Sheldrick, 2008 ▶); molecular graphics: *OLEX2* (Dolomanov *et al.*, 2009 ▶); software used to prepare material for publication: *OLEX2* and *publCIF* (Westrip, 2010 ▶).

## Supplementary Material

Crystal structure: contains datablock(s) I, New_Global_Publ_Block. DOI: 10.1107/S1600536814021357/hg5407sup1.cif

Structure factors: contains datablock(s) I. DOI: 10.1107/S1600536814021357/hg5407Isup2.hkl

**Fig. 1.**
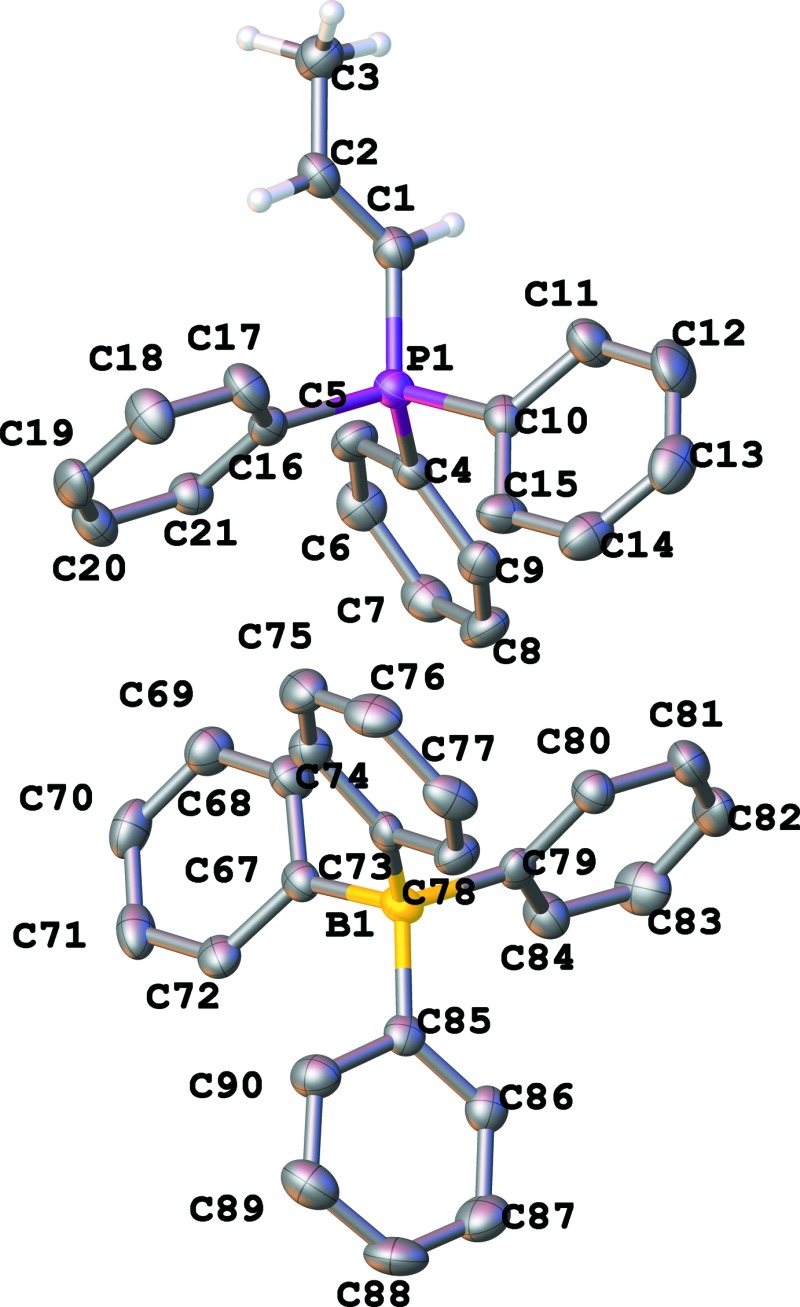
A ball-and-stick representaion of the structure of I. Only one of the two cation/anion pairs are shown. Hydrogen atoms on the aromatic rings have been removed for clarity.

I . DOI: 10.1107/S1600536814021357/hg5407fig1.tif
A ball-and-stick representaion of the structure of **I**. Only one of the two cation/anion pairs are shown. Hydrogen atoms on the aromatic rings have been removed for clarity.

**Fig. 2.**
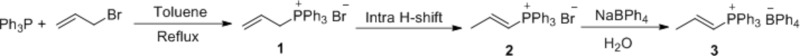
Reaction scheme for the synthesis of I.

I . DOI: 10.1107/S1600536814021357/hg5407fig2.tif
Reaction scheme for the synthesis of **I**.

CCDC reference: 1026273

Additional supporting information:  crystallographic information; 3D view; checkCIF report

## supplementary crystallographic information

### S1. Chemical Context

In recent years, phospho­rus-carbon bond-forming reactions have been the object of inter­est in the modern organo­phospho­rus chemistry due to the importance of these products in a variety of fields ranging from material science to the synthesis of biologically active molecules. Phospho­nium salts have been known since the 1980s; however, they were rarely employed until the 1990s, when phosphines were used as precursors on the large scale, see: Bellina *et al.* (2012).

Phospho­nium-based ionic liquids emerged as a very promising alternative to imidazolium analogs due to their superior thermal stability and inertness in the basic environment. The presence of an acidic hydrogen at the C2 position of imidazolium rings in many ionic liquids, leads to the thermal instability that limits the applications of these inter­esting organic fluids, see: Chowdhury *et al.* (2007).

In this study, we found that the vinyl­tri­phenyl­phospho­nium bromide could be synthesized *via* the nucleophilic attack of tri­phenyl­phosphine to allyl bromide to form the corresponding betaine 1, followed by the intra-hydrogen shift reaction (Figure 2). In the next step, metathesis reaction of vinyl­triphenyphospho­nium bromide 2 with sodium tetra­phenyl­borate led to the formation vinyl­triphenyphospho­nium tetra­phenyl­borane 3 (*T*_m_= 72–74° C) as the final product in high yield (78%).

### S2. Experimental

Triphenylphosphine (0.5 mmol) and allyl bromide (0.7 mmol) were dissolved in toluene (2.0 ml) and the mixture refluxed for 48 h. The solvent was removed in a vacuum and the crude product was dissolved in 4.0 ml of H_2_O. Then, a solution of sodium tetraphenylborate (0.6 mmol) in 2.0 ml of H_2_O was added to the original solution of crude product and was stirred for 24 h. The final product was separated *via* filtration and washed with H_2_O (3 *x* 5 ml) to yield white crystals of the title complex.

### S3. Refinement

H-atoms were placed in calculated positions and allowed to ride during subsequent refinement with *U*_iso_(H) = 1.2*U*_eq_(C) and C—H distances of 0.93 Å, except for the methyl H atoms which had *U*_iso_(H) = 1.5*U*_eq_(C) and C—H distances of 0.96 Å.

### Crystal data

**Table:**

C_21_H_20_P^+^·C_24_H_20_B^−^	*F*(000) = 1320
*M**_r_* = 622.55	*D*_x_ = 1.158 Mg m^−^^3^
Monoclinic, *P*2_1_	Mo *K*α radiation, λ = 0.7107 Å
*a* = 9.2752 (4) Å	Cell parameters from 4486 reflections
*b* = 35.7838 (15) Å	θ = 3.0–25.0°
*c* = 10.9515 (5) Å	µ = 0.11 mm^−^^1^
β = 100.721 (4)°	*T* = 180 K
*V* = 3571.4 (3) Å^3^	Prism, colourless
*Z* = 4	0.43 × 0.18 × 0.17 mm

### Data collection

**Table:**

Agilent Xcalibur, Eos diffractometer	12797 independent reflections
Radiation source: Enhance (Mo) X-ray Source	9444 reflections with *I* > 2σ(*I*)
Graphite monochromator	*R*_int_ = 0.038
Detector resolution: 16.0514 pixels mm^-1^	θ_max_ = 25.2°, θ_min_ = 2.5°
ω scans	*h* = −9→11
Absorption correction: multi-scan (*CrysAlis PRO*; Agilent, 2014)	*k* = −42→42
*T*_min_ = 0.958, *T*_max_ = 1.000	*l* = −13→12
24267 measured reflections	

### Refinement

**Table:**

Refinement on *F*^2^	Secondary atom site location: difference Fourier map
Least-squares matrix: full	Hydrogen site location: inferred from neighbouring sites
*R*[*F*^2^ > 2σ(*F*^2^)] = 0.058	H-atom parameters constrained
*wR*(*F*^2^) = 0.105	*w* = 1/[σ^2^(*F*_o_^2^) + (0.038*P*)^2^] where *P* = (*F*_o_^2^ + 2*F*_c_^2^)/3
*S* = 1.01	(Δ/σ)_max_ = 0.002
12797 reflections	Δρ_max_ = 0.35 e Å^−^^3^
849 parameters	Δρ_min_ = −0.23 e Å^−^^3^
1 restraint	Absolute structure: Flack (1983)
Primary atom site location: structure-invariant direct methods	Absolute structure parameter: 0.02 (7)

### Special details

**Table:**

Geometry. All e.s.d.'s (except the e.s.d. in the dihedral angle between two l.s. planes) are estimated using the full covariance matrix. The cell e.s.d.'s are taken into account individually in the estimation of e.s.d.'s in distances, angles and torsion angles; correlations between e.s.d.'s in cell parameters are only used when they are defined by crystal symmetry. An approximate (isotropic) treatment of cell e.s.d.'s is used for estimating e.s.d.'s involving l.s. planes.
Refinement. Refinement of *F*^2^ against ALL reflections. The weighted *R*-factor *wR* and goodness of fit *S* are based on *F*^2^, conventional *R*-factors *R* are based on *F*, with *F* set to zero for negative *F*^2^. The threshold expression of *F*^2^ > 2σ(*F*^2^) is used only for calculating *R*-factors(gt) *etc*. and is not relevant to the choice of reflections for refinement. *R*-factors based on *F*^2^ are statistically about twice as large as those based on *F*, and *R*- factors based on ALL data will be even larger.

### Fractional atomic coordinates and isotropic or equivalent isotropic displacement parameters (Å^2^)

**Table:**

	*x*	*y*	*z*	*U*_iso_*/*U*_eq_	
P1	0.34715 (9)	0.43752 (2)	0.03996 (8)	0.0268 (2)	
B1	0.2156 (4)	0.36038 (10)	0.5307 (3)	0.0258 (9)	
C72	0.4956 (3)	0.35850 (9)	0.6594 (3)	0.0304 (8)	
H72	0.4650	0.3427	0.7170	0.037*	
C17	0.4142 (4)	0.36574 (9)	−0.0218 (3)	0.0359 (9)	
H17	0.3380	0.3686	−0.0896	0.043*	
C79	0.1227 (3)	0.39980 (9)	0.5080 (3)	0.0257 (7)	
C2	0.4719 (4)	0.45131 (9)	−0.1653 (3)	0.0315 (8)	
H2	0.5098	0.4273	−0.1534	0.038*	
C4	0.3897 (3)	0.46572 (9)	0.1772 (3)	0.0255 (8)	
C18	0.4917 (4)	0.33265 (9)	−0.0053 (3)	0.0398 (9)	
H18	0.4685	0.3135	−0.0628	0.048*	
C73	0.1727 (3)	0.33367 (8)	0.4059 (3)	0.0252 (8)	
C67	0.3916 (3)	0.37116 (8)	0.5581 (3)	0.0260 (8)	
C21	0.5619 (3)	0.38947 (9)	0.1647 (3)	0.0305 (8)	
H21	0.5861	0.4086	0.2223	0.037*	
C16	0.4499 (3)	0.39493 (9)	0.0632 (3)	0.0256 (8)	
C11	0.0563 (4)	0.44317 (10)	−0.0802 (3)	0.0388 (9)	
H11	0.0878	0.4615	−0.1296	0.047*	
C10	0.1559 (3)	0.42514 (9)	0.0120 (3)	0.0281 (8)	
C5	0.5204 (4)	0.48620 (9)	0.1970 (3)	0.0334 (8)	
H5	0.5801	0.4860	0.1375	0.040*	
C75	0.2179 (4)	0.30267 (10)	0.2179 (3)	0.0392 (9)	
H75	0.2819	0.2979	0.1637	0.047*	
C9	0.2993 (4)	0.46700 (9)	0.2647 (3)	0.0300 (8)	
H9	0.2104	0.4543	0.2509	0.036*	
C68	0.4482 (4)	0.39505 (9)	0.4765 (3)	0.0340 (8)	
H68	0.3841	0.4042	0.4074	0.041*	
C80	0.0193 (3)	0.40884 (9)	0.4016 (3)	0.0310 (8)	
H80	−0.0006	0.3913	0.3380	0.037*	
C87	0.0463 (4)	0.32577 (11)	0.8201 (3)	0.0449 (10)	
H87	−0.0176	0.3347	0.8695	0.054*	
C14	−0.0395 (4)	0.38877 (11)	0.0646 (3)	0.0414 (10)	
H14	−0.0719	0.3701	0.1121	0.050*	
C1	0.3916 (3)	0.46345 (9)	−0.0853 (3)	0.0297 (8)	
H1	0.3555	0.4877	−0.0959	0.036*	
C83	0.0698 (4)	0.46182 (10)	0.5853 (3)	0.0443 (10)	
H83	0.0869	0.4794	0.6489	0.053*	
C70	0.6933 (4)	0.39238 (10)	0.5945 (3)	0.0386 (9)	
H70	0.7918	0.3992	0.6068	0.046*	
C84	0.1452 (4)	0.42801 (9)	0.5987 (3)	0.0345 (9)	
H84	0.2136	0.4239	0.6711	0.041*	
C77	−0.0138 (4)	0.29464 (9)	0.2771 (3)	0.0355 (9)	
H77	−0.1074	0.2843	0.2633	0.043*	
C74	0.2627 (4)	0.32536 (9)	0.3213 (3)	0.0322 (8)	
H74	0.3569	0.3354	0.3343	0.039*	
C6	0.5614 (4)	0.50689 (9)	0.3053 (3)	0.0354 (9)	
H6	0.6488	0.5203	0.3184	0.043*	
C85	0.1718 (3)	0.33667 (9)	0.6475 (3)	0.0264 (8)	
C3	0.5066 (4)	0.47268 (9)	−0.2720 (3)	0.0374 (9)	
H3A	0.4727	0.4591	−0.3474	0.056*	
H3B	0.6107	0.4762	−0.2612	0.056*	
H3C	0.4587	0.4966	−0.2764	0.056*	
C81	−0.0547 (4)	0.44277 (10)	0.3868 (3)	0.0393 (9)	
H81	−0.1216	0.4475	0.3140	0.047*	
C19	0.6038 (4)	0.32777 (10)	0.0965 (3)	0.0374 (9)	
H19	0.6555	0.3054	0.1073	0.045*	
C13	−0.1381 (4)	0.40692 (11)	−0.0248 (4)	0.0450 (10)	
H13	−0.2374	0.4011	−0.0359	0.054*	
C69	0.5953 (4)	0.40575 (10)	0.4934 (3)	0.0377 (9)	
H69	0.6270	0.4218	0.4370	0.045*	
C90	0.2321 (4)	0.30060 (9)	0.6724 (3)	0.0355 (9)	
H90	0.2948	0.2914	0.6223	0.043*	
C82	−0.0297 (4)	0.46938 (10)	0.4787 (4)	0.0412 (10)	
H82	−0.0793	0.4921	0.4690	0.049*	
C15	0.1073 (4)	0.39795 (10)	0.0846 (3)	0.0346 (9)	
H15	0.1733	0.3859	0.1465	0.042*	
C78	0.0317 (4)	0.31736 (9)	0.3794 (3)	0.0311 (8)	
H78	−0.0333	0.3221	0.4328	0.037*	
C8	0.3430 (4)	0.48758 (9)	0.3735 (3)	0.0367 (9)	
H8	0.2840	0.4879	0.4335	0.044*	
C88	0.1060 (4)	0.29117 (11)	0.8424 (3)	0.0476 (11)	
H88	0.0832	0.2764	0.9060	0.057*	
C89	0.2021 (4)	0.27830 (10)	0.7679 (3)	0.0444 (10)	
H89	0.2458	0.2549	0.7822	0.053*	
C20	0.6382 (4)	0.35622 (10)	0.1817 (3)	0.0375 (9)	
H20	0.7127	0.3530	0.2505	0.045*	
C7	0.4730 (4)	0.50756 (9)	0.3932 (3)	0.0376 (9)	
H7	0.5005	0.5214	0.4656	0.045*	
C86	0.0791 (3)	0.34815 (9)	0.7245 (3)	0.0311 (8)	
H86	0.0366	0.3717	0.7124	0.037*	
C76	0.0797 (4)	0.28730 (9)	0.1955 (3)	0.0389 (9)	
H76	0.0495	0.2722	0.1264	0.047*	
C71	0.6427 (4)	0.36872 (10)	0.6768 (3)	0.0388 (9)	
H71	0.7080	0.3595	0.7450	0.047*	
C12	−0.0905 (4)	0.43336 (12)	−0.0969 (3)	0.0494 (10)	
H12	−0.1577	0.4451	−0.1587	0.059*	
P2	0.08786 (9)	0.68072 (2)	0.54835 (8)	0.0300 (2)	
C25	0.1647 (4)	0.64615 (9)	0.4583 (3)	0.0310 (8)	
C37	−0.1002 (3)	0.68908 (9)	0.4796 (3)	0.0301 (8)	
C31	0.1892 (3)	0.72348 (9)	0.5475 (3)	0.0286 (8)	
C35	0.3943 (4)	0.76235 (10)	0.6324 (3)	0.0385 (9)	
H35	0.4766	0.7671	0.6932	0.046*	
C36	0.3151 (4)	0.72971 (9)	0.6382 (3)	0.0323 (8)	
H36	0.3449	0.7123	0.7009	0.039*	
C26	0.1572 (4)	0.60903 (10)	0.4903 (3)	0.0380 (9)	
H26	0.1120	0.6020	0.5558	0.046*	
C32	0.1483 (4)	0.74896 (10)	0.4528 (3)	0.0393 (9)	
H32	0.0663	0.7445	0.3915	0.047*	
C23	−0.0020 (4)	0.65275 (10)	0.7555 (3)	0.0387 (9)	
H23	−0.0964	0.6542	0.7087	0.046*	
C42	−0.1630 (4)	0.67238 (9)	0.3679 (3)	0.0358 (9)	
H42	−0.1082	0.6559	0.3293	0.043*	
C34	0.3532 (4)	0.78776 (10)	0.5384 (4)	0.0433 (10)	
H34	0.4084	0.8093	0.5348	0.052*	
C41	−0.3067 (4)	0.68013 (11)	0.3140 (4)	0.0479 (10)	
H41	−0.3489	0.6690	0.2391	0.057*	
C38	−0.1820 (4)	0.71415 (10)	0.5365 (3)	0.0416 (10)	
H38	−0.1393	0.7261	0.6097	0.050*	
C29	0.2864 (4)	0.62943 (12)	0.2914 (4)	0.0542 (11)	
H29	0.3294	0.6361	0.2243	0.065*	
C40	−0.3871 (4)	0.70459 (11)	0.3721 (4)	0.0507 (11)	
H40	−0.4835	0.7100	0.3356	0.061*	
C22	0.1060 (4)	0.66368 (9)	0.7023 (3)	0.0365 (9)	
H22	0.2003	0.6624	0.7493	0.044*	
C24	0.0135 (4)	0.63812 (11)	0.8853 (3)	0.0492 (10)	
H24A	−0.0184	0.6568	0.9373	0.074*	
H24B	0.1145	0.6321	0.9166	0.074*	
H24C	−0.0455	0.6161	0.8854	0.074*	
C30	0.2276 (4)	0.65675 (11)	0.3583 (3)	0.0449 (10)	
H30	0.2304	0.6818	0.3362	0.054*	
C33	0.2303 (4)	0.78124 (10)	0.4498 (4)	0.0465 (10)	
H33	0.2017	0.7987	0.3870	0.056*	
C28	0.2806 (4)	0.59237 (12)	0.3250 (4)	0.0511 (11)	
H28	0.3195	0.5741	0.2802	0.061*	
C39	−0.3264 (4)	0.72109 (11)	0.4838 (4)	0.0507 (11)	
H39	−0.3827	0.7369	0.5234	0.061*	
C27	0.2180 (4)	0.58236 (10)	0.4235 (3)	0.0430 (10)	
H27	0.2162	0.5573	0.4460	0.052*	
C61	0.4167 (3)	0.57468 (8)	0.8922 (3)	0.0272 (8)	
C52	0.9188 (4)	0.54378 (10)	1.1853 (3)	0.0393 (9)	
H52	0.9912	0.5326	1.2441	0.047*	
C54	0.7219 (4)	0.53955 (9)	1.0089 (3)	0.0342 (9)	
H54	0.6627	0.5246	0.9506	0.041*	
C66	0.3161 (4)	0.56711 (9)	0.7820 (3)	0.0323 (8)	
H66	0.3429	0.5725	0.7061	0.039*	
C50	0.7902 (4)	0.59831 (10)	1.0974 (3)	0.0360 (9)	
H50	0.7788	0.6241	1.0998	0.043*	
C43	0.5464 (4)	0.64126 (9)	0.9268 (3)	0.0330 (9)	
C51	0.8976 (4)	0.58191 (10)	1.1854 (3)	0.0421 (10)	
H51	0.9558	0.5967	1.2450	0.051*	
C55	0.6257 (3)	0.59416 (9)	0.7602 (3)	0.0335 (9)	
C49	0.6979 (3)	0.57829 (9)	1.0051 (3)	0.0257 (8)	
C56	0.7273 (4)	0.56889 (10)	0.7309 (3)	0.0389 (9)	
H56	0.7713	0.5525	0.7924	0.047*	
C63	0.2317 (4)	0.54926 (9)	1.0030 (3)	0.0359 (9)	
H63	0.2048	0.5432	1.0783	0.043*	
C62	0.3697 (4)	0.56437 (9)	1.0006 (3)	0.0322 (8)	
H62	0.4338	0.5677	1.0758	0.039*	
C60	0.5663 (4)	0.61744 (11)	0.6606 (4)	0.0464 (10)	
H60	0.4968	0.6350	0.6735	0.056*	
C53	0.8306 (4)	0.52248 (10)	1.0962 (3)	0.0360 (9)	
H53	0.8437	0.4967	1.0944	0.043*	
C48	0.6406 (4)	0.66971 (10)	0.8992 (4)	0.0547 (12)	
H48	0.7171	0.6632	0.8592	0.066*	
C44	0.4331 (4)	0.65409 (10)	0.9846 (3)	0.0412 (10)	
H44	0.3671	0.6367	1.0054	0.049*	
C59	0.6042 (5)	0.61595 (13)	0.5444 (4)	0.0584 (12)	
H59	0.5602	0.6322	0.4821	0.070*	
C57	0.7675 (4)	0.56666 (12)	0.6137 (4)	0.0511 (11)	
H57	0.8355	0.5489	0.5991	0.061*	
C58	0.7067 (5)	0.59057 (13)	0.5208 (4)	0.0535 (12)	
H58	0.7342	0.5897	0.4435	0.064*	
C65	0.1788 (4)	0.55201 (9)	0.7824 (4)	0.0389 (9)	
H65	0.1152	0.5477	0.7075	0.047*	
B2	0.5727 (4)	0.59688 (10)	0.8955 (4)	0.0293 (9)	
C64	0.1356 (4)	0.54337 (9)	0.8924 (4)	0.0431 (10)	
H64	0.0426	0.5337	0.8924	0.052*	
C46	0.5112 (6)	0.71806 (12)	0.9865 (4)	0.0648 (14)	
H46	0.5007	0.7430	1.0073	0.078*	
C45	0.4144 (5)	0.69151 (11)	1.0126 (4)	0.0557 (12)	
H45	0.3358	0.6986	1.0492	0.067*	
C47	0.6238 (5)	0.70708 (11)	0.9292 (5)	0.0684 (14)	
H47	0.6896	0.7248	0.9101	0.082*	

### Atomic displacement parameters (Å^2^)

**Table:**

	*U*^11^	*U*^22^	*U*^33^	*U*^12^	*U*^13^	*U*^23^
P1	0.0277 (5)	0.0279 (5)	0.0242 (5)	0.0009 (4)	0.0031 (4)	0.0011 (4)
B1	0.031 (2)	0.026 (2)	0.020 (2)	0.0004 (17)	0.0006 (17)	−0.0013 (16)
C72	0.034 (2)	0.033 (2)	0.0247 (19)	0.0045 (16)	0.0052 (16)	−0.0060 (15)
C17	0.035 (2)	0.034 (2)	0.034 (2)	0.0030 (17)	−0.0053 (17)	0.0023 (17)
C79	0.0202 (17)	0.0269 (18)	0.031 (2)	−0.0013 (14)	0.0073 (15)	0.0021 (15)
C2	0.032 (2)	0.0285 (19)	0.032 (2)	0.0037 (15)	0.0003 (17)	0.0031 (15)
C4	0.0269 (19)	0.0253 (17)	0.0236 (19)	0.0021 (15)	0.0026 (15)	0.0024 (14)
C18	0.048 (2)	0.027 (2)	0.042 (2)	0.0048 (18)	0.0020 (19)	−0.0076 (17)
C73	0.0252 (19)	0.0228 (18)	0.0269 (19)	0.0067 (14)	0.0026 (15)	0.0020 (14)
C67	0.0287 (19)	0.0239 (18)	0.0247 (19)	0.0046 (14)	0.0031 (15)	−0.0025 (15)
C21	0.032 (2)	0.033 (2)	0.025 (2)	0.0005 (16)	0.0026 (16)	−0.0017 (15)
C16	0.0218 (17)	0.0280 (18)	0.027 (2)	−0.0023 (14)	0.0056 (15)	0.0015 (15)
C11	0.035 (2)	0.042 (2)	0.037 (2)	0.0031 (17)	−0.0017 (17)	0.0072 (17)
C10	0.0266 (19)	0.032 (2)	0.0239 (19)	0.0042 (15)	0.0007 (15)	−0.0057 (15)
C5	0.031 (2)	0.038 (2)	0.033 (2)	−0.0032 (17)	0.0121 (16)	0.0027 (17)
C75	0.046 (2)	0.042 (2)	0.031 (2)	0.0094 (19)	0.0120 (18)	−0.0022 (17)
C9	0.032 (2)	0.0237 (18)	0.035 (2)	0.0003 (15)	0.0079 (17)	0.0025 (16)
C68	0.031 (2)	0.039 (2)	0.030 (2)	0.0005 (17)	0.0008 (16)	0.0018 (16)
C80	0.033 (2)	0.030 (2)	0.029 (2)	0.0030 (16)	0.0044 (16)	−0.0008 (15)
C87	0.049 (3)	0.057 (3)	0.031 (2)	0.001 (2)	0.0119 (19)	−0.007 (2)
C14	0.038 (2)	0.052 (2)	0.036 (2)	−0.0099 (19)	0.0106 (18)	−0.0074 (19)
C1	0.030 (2)	0.0288 (19)	0.030 (2)	0.0032 (16)	0.0027 (16)	−0.0011 (16)
C83	0.061 (3)	0.030 (2)	0.043 (2)	0.0023 (19)	0.011 (2)	−0.0076 (18)
C70	0.0238 (19)	0.044 (2)	0.049 (2)	−0.0023 (17)	0.0074 (18)	−0.0164 (19)
C84	0.038 (2)	0.031 (2)	0.033 (2)	0.0025 (16)	0.0015 (17)	−0.0002 (16)
C77	0.038 (2)	0.033 (2)	0.031 (2)	−0.0114 (17)	−0.0046 (18)	0.0031 (17)
C74	0.030 (2)	0.033 (2)	0.033 (2)	0.0004 (16)	0.0047 (17)	−0.0003 (16)
C6	0.037 (2)	0.030 (2)	0.038 (2)	−0.0052 (16)	0.0014 (18)	−0.0045 (17)
C85	0.0246 (18)	0.0309 (19)	0.0216 (18)	−0.0014 (15)	−0.0014 (15)	−0.0051 (15)
C3	0.039 (2)	0.037 (2)	0.038 (2)	0.0008 (17)	0.0118 (18)	−0.0001 (17)
C81	0.035 (2)	0.045 (2)	0.037 (2)	0.0111 (18)	0.0035 (17)	0.0107 (19)
C19	0.039 (2)	0.028 (2)	0.047 (2)	0.0123 (17)	0.0117 (19)	0.0083 (18)
C13	0.029 (2)	0.061 (3)	0.043 (3)	−0.003 (2)	0.0015 (19)	−0.017 (2)
C69	0.033 (2)	0.042 (2)	0.038 (2)	−0.0047 (17)	0.0073 (18)	−0.0022 (17)
C90	0.043 (2)	0.034 (2)	0.030 (2)	0.0001 (17)	0.0080 (17)	−0.0016 (16)
C82	0.044 (2)	0.034 (2)	0.048 (3)	0.0133 (18)	0.015 (2)	0.0103 (19)
C15	0.034 (2)	0.043 (2)	0.026 (2)	−0.0069 (17)	0.0032 (16)	0.0048 (17)
C78	0.029 (2)	0.040 (2)	0.026 (2)	−0.0026 (16)	0.0080 (16)	−0.0031 (16)
C8	0.045 (2)	0.037 (2)	0.033 (2)	0.0038 (18)	0.0195 (18)	0.0007 (17)
C88	0.067 (3)	0.051 (3)	0.025 (2)	−0.004 (2)	0.009 (2)	0.0064 (18)
C89	0.056 (3)	0.037 (2)	0.036 (2)	0.0000 (19)	−0.002 (2)	0.0040 (18)
C20	0.034 (2)	0.039 (2)	0.036 (2)	0.0033 (17)	−0.0021 (17)	0.0086 (18)
C7	0.049 (2)	0.034 (2)	0.028 (2)	0.0055 (18)	0.0008 (18)	−0.0053 (16)
C86	0.0281 (19)	0.033 (2)	0.031 (2)	−0.0005 (16)	0.0023 (16)	−0.0054 (16)
C76	0.057 (3)	0.035 (2)	0.023 (2)	−0.0019 (18)	0.0044 (19)	−0.0067 (16)
C71	0.029 (2)	0.049 (2)	0.034 (2)	0.0108 (18)	−0.0059 (17)	−0.0131 (18)
C12	0.033 (2)	0.065 (3)	0.044 (2)	0.011 (2)	−0.0089 (19)	0.002 (2)
P2	0.0279 (5)	0.0312 (5)	0.0305 (5)	0.0010 (4)	0.0041 (4)	−0.0007 (4)
C25	0.0248 (19)	0.038 (2)	0.028 (2)	0.0019 (16)	−0.0026 (15)	−0.0018 (16)
C37	0.0265 (18)	0.027 (2)	0.036 (2)	0.0002 (15)	0.0057 (16)	0.0015 (16)
C31	0.0210 (18)	0.0307 (19)	0.035 (2)	0.0038 (15)	0.0072 (15)	−0.0030 (16)
C35	0.028 (2)	0.047 (2)	0.041 (2)	−0.0085 (18)	0.0077 (17)	−0.0137 (19)
C36	0.030 (2)	0.037 (2)	0.031 (2)	0.0030 (16)	0.0076 (16)	−0.0026 (16)
C26	0.040 (2)	0.041 (2)	0.030 (2)	0.0040 (18)	−0.0012 (17)	−0.0020 (17)
C32	0.038 (2)	0.037 (2)	0.041 (2)	−0.0008 (18)	0.0032 (18)	0.0007 (18)
C23	0.036 (2)	0.048 (2)	0.033 (2)	−0.0026 (18)	0.0068 (18)	−0.0014 (18)
C42	0.031 (2)	0.033 (2)	0.041 (2)	0.0024 (16)	0.0033 (17)	−0.0003 (17)
C34	0.038 (2)	0.040 (2)	0.054 (3)	−0.0076 (18)	0.015 (2)	−0.005 (2)
C41	0.038 (2)	0.046 (2)	0.054 (3)	−0.008 (2)	−0.006 (2)	0.008 (2)
C38	0.042 (2)	0.039 (2)	0.046 (2)	−0.0017 (18)	0.014 (2)	−0.0011 (18)
C29	0.048 (3)	0.072 (3)	0.046 (3)	0.001 (2)	0.020 (2)	−0.011 (2)
C40	0.021 (2)	0.053 (3)	0.076 (3)	−0.0001 (19)	0.005 (2)	0.022 (2)
C22	0.030 (2)	0.040 (2)	0.039 (2)	−0.0026 (17)	0.0044 (17)	0.0001 (17)
C24	0.043 (2)	0.058 (3)	0.048 (3)	0.000 (2)	0.013 (2)	0.005 (2)
C30	0.048 (2)	0.040 (2)	0.050 (3)	−0.0008 (19)	0.018 (2)	−0.0051 (19)
C33	0.044 (2)	0.043 (2)	0.053 (3)	0.003 (2)	0.013 (2)	0.0112 (19)
C28	0.054 (3)	0.049 (3)	0.048 (3)	0.015 (2)	0.003 (2)	−0.010 (2)
C39	0.040 (3)	0.047 (2)	0.072 (3)	0.012 (2)	0.026 (2)	0.007 (2)
C27	0.052 (3)	0.036 (2)	0.036 (2)	0.0117 (19)	−0.005 (2)	−0.0047 (18)
C61	0.0252 (18)	0.0180 (17)	0.038 (2)	−0.0001 (14)	0.0045 (16)	−0.0008 (15)
C52	0.031 (2)	0.051 (2)	0.034 (2)	0.0045 (18)	−0.0019 (17)	0.0094 (18)
C54	0.030 (2)	0.035 (2)	0.035 (2)	−0.0058 (16)	0.0012 (17)	0.0032 (17)
C66	0.033 (2)	0.0299 (19)	0.033 (2)	−0.0006 (16)	0.0036 (16)	0.0003 (16)
C50	0.034 (2)	0.038 (2)	0.035 (2)	0.0038 (17)	0.0039 (17)	−0.0023 (17)
C43	0.0250 (19)	0.032 (2)	0.037 (2)	−0.0024 (16)	−0.0074 (16)	0.0071 (16)
C51	0.039 (2)	0.050 (2)	0.032 (2)	0.0047 (18)	−0.0082 (18)	−0.0082 (18)
C55	0.0256 (19)	0.038 (2)	0.034 (2)	−0.0118 (16)	−0.0036 (16)	0.0086 (17)
C49	0.0208 (17)	0.0309 (19)	0.0266 (19)	−0.0031 (14)	0.0073 (15)	0.0010 (15)
C56	0.038 (2)	0.043 (2)	0.034 (2)	−0.0038 (18)	0.0028 (18)	−0.0019 (17)
C63	0.035 (2)	0.028 (2)	0.049 (3)	0.0037 (16)	0.0183 (19)	0.0032 (17)
C62	0.032 (2)	0.0278 (19)	0.037 (2)	0.0014 (15)	0.0070 (17)	0.0012 (16)
C60	0.037 (2)	0.059 (3)	0.041 (3)	−0.0031 (19)	0.0003 (19)	0.015 (2)
C53	0.034 (2)	0.033 (2)	0.043 (2)	0.0011 (17)	0.0120 (18)	0.0086 (17)
C48	0.040 (2)	0.038 (2)	0.084 (3)	−0.0065 (18)	0.008 (2)	0.015 (2)
C44	0.047 (2)	0.034 (2)	0.038 (2)	0.0004 (18)	−0.0060 (19)	−0.0025 (17)
C59	0.059 (3)	0.074 (3)	0.038 (3)	−0.016 (3)	0.001 (2)	0.024 (2)
C57	0.045 (2)	0.067 (3)	0.042 (3)	−0.015 (2)	0.009 (2)	−0.014 (2)
C58	0.061 (3)	0.072 (3)	0.027 (2)	−0.031 (2)	0.007 (2)	−0.001 (2)
C65	0.029 (2)	0.030 (2)	0.052 (3)	−0.0008 (16)	−0.0078 (19)	−0.0001 (18)
B2	0.027 (2)	0.026 (2)	0.034 (2)	−0.0027 (17)	0.0029 (18)	0.0035 (18)
C64	0.025 (2)	0.031 (2)	0.074 (3)	−0.0004 (16)	0.012 (2)	0.004 (2)
C46	0.076 (4)	0.033 (3)	0.070 (3)	0.006 (3)	−0.024 (3)	−0.005 (2)
C45	0.061 (3)	0.040 (3)	0.060 (3)	0.009 (2)	−0.004 (2)	−0.010 (2)
C47	0.066 (3)	0.030 (3)	0.099 (4)	−0.013 (2)	−0.010 (3)	0.018 (2)

### Geometric parameters (Å, º)

**Table:**

P1—C4	1.792 (3)	P2—C25	1.809 (3)
P1—C16	1.791 (3)	P2—C37	1.792 (3)
P1—C10	1.798 (3)	P2—C31	1.797 (3)
P1—C1	1.767 (3)	P2—C22	1.771 (4)
B1—C79	1.648 (5)	C25—C26	1.379 (5)
B1—C73	1.655 (5)	C25—C30	1.386 (5)
B1—C67	1.650 (5)	C37—C42	1.389 (4)
B1—C85	1.647 (5)	C37—C38	1.394 (4)
C72—H72	0.9300	C31—C36	1.403 (4)
C72—C67	1.403 (4)	C31—C32	1.380 (4)
C72—C71	1.391 (4)	C35—H35	0.9300
C17—H17	0.9300	C35—C36	1.387 (5)
C17—C18	1.380 (4)	C35—C34	1.374 (5)
C17—C16	1.397 (4)	C36—H36	0.9300
C79—C80	1.403 (4)	C26—H26	0.9300
C79—C84	1.404 (4)	C26—C27	1.385 (5)
C2—H2	0.9300	C32—H32	0.9300
C2—C1	1.324 (5)	C32—C33	1.387 (5)
C2—C3	1.481 (4)	C23—H23	0.9300
C4—C5	1.398 (4)	C23—C22	1.309 (5)
C4—C9	1.386 (4)	C23—C24	1.497 (5)
C18—H18	0.9300	C42—H42	0.9300
C18—C19	1.387 (5)	C42—C41	1.382 (4)
C73—C74	1.390 (4)	C34—H34	0.9300
C73—C78	1.413 (4)	C34—C33	1.372 (5)
C67—C68	1.407 (4)	C41—H41	0.9300
C21—H21	0.9300	C41—C40	1.380 (5)
C21—C16	1.386 (4)	C38—H38	0.9300
C21—C20	1.379 (4)	C38—C39	1.379 (5)
C11—H11	0.9300	C29—H29	0.9300
C11—C10	1.394 (4)	C29—C30	1.393 (5)
C11—C12	1.385 (5)	C29—C28	1.380 (5)
C10—C15	1.384 (4)	C40—H40	0.9300
C5—H5	0.9300	C40—C39	1.381 (5)
C5—C6	1.390 (4)	C22—H22	0.9300
C75—H75	0.9300	C24—H24A	0.9600
C75—C74	1.393 (4)	C24—H24B	0.9600
C75—C76	1.375 (5)	C24—H24C	0.9600
C9—H9	0.9300	C30—H30	0.9300
C9—C8	1.396 (4)	C33—H33	0.9300
C68—H68	0.9300	C28—H28	0.9300
C68—C69	1.395 (4)	C28—C27	1.364 (5)
C80—H80	0.9300	C39—H39	0.9300
C80—C81	1.389 (4)	C27—H27	0.9300
C87—H87	0.9300	C61—C66	1.408 (4)
C87—C88	1.360 (5)	C61—C62	1.389 (4)
C87—C86	1.396 (5)	C61—B2	1.644 (5)
C14—H14	0.9300	C52—H52	0.9300
C14—C13	1.373 (5)	C52—C51	1.378 (5)
C14—C15	1.377 (4)	C52—C53	1.380 (5)
C1—H1	0.9300	C54—H54	0.9300
C83—H83	0.9300	C54—C49	1.403 (4)
C83—C84	1.392 (5)	C54—C53	1.394 (4)
C83—C82	1.374 (5)	C66—H66	0.9300
C70—H70	0.9300	C66—C65	1.384 (4)
C70—C69	1.381 (5)	C50—H50	0.9300
C70—C71	1.382 (5)	C50—C51	1.381 (4)
C84—H84	0.9300	C50—C49	1.395 (4)
C77—H77	0.9300	C43—C48	1.410 (5)
C77—C78	1.385 (4)	C43—C44	1.401 (5)
C77—C76	1.381 (5)	C43—B2	1.652 (5)
C74—H74	0.9300	C51—H51	0.9300
C6—H6	0.9300	C55—C56	1.386 (5)
C6—C7	1.375 (5)	C55—C60	1.401 (4)
C85—C90	1.413 (4)	C55—B2	1.649 (5)
C85—C86	1.374 (4)	C49—B2	1.648 (5)
C3—H3A	0.9600	C56—H56	0.9300
C3—H3B	0.9600	C56—C57	1.404 (5)
C3—H3C	0.9600	C63—H63	0.9300
C81—H81	0.9300	C63—C62	1.394 (4)
C81—C82	1.374 (5)	C63—C64	1.380 (5)
C19—H19	0.9300	C62—H62	0.9300
C19—C20	1.377 (5)	C60—H60	0.9300
C13—H13	0.9300	C60—C59	1.383 (5)
C13—C12	1.358 (5)	C53—H53	0.9300
C69—H69	0.9300	C48—H48	0.9300
C90—H90	0.9300	C48—C47	1.392 (5)
C90—C89	1.384 (5)	C44—H44	0.9300
C82—H82	0.9300	C44—C45	1.392 (5)
C15—H15	0.9300	C59—H59	0.9300
C78—H78	0.9300	C59—C58	1.374 (6)
C8—H8	0.9300	C57—H57	0.9300
C8—C7	1.384 (5)	C57—C58	1.367 (5)
C88—H88	0.9300	C58—H58	0.9300
C88—C89	1.394 (5)	C65—H65	0.9300
C89—H89	0.9300	C65—C64	1.374 (5)
C20—H20	0.9300	C64—H64	0.9300
C7—H7	0.9300	C46—H46	0.9300
C86—H86	0.9300	C46—C45	1.373 (6)
C76—H76	0.9300	C46—C47	1.372 (6)
C71—H71	0.9300	C45—H45	0.9300
C12—H12	0.9300	C47—H47	0.9300
			
C4—P1—C10	109.76 (15)	C37—P2—C25	109.67 (15)
C16—P1—C4	109.36 (14)	C37—P2—C31	108.98 (14)
C16—P1—C10	107.26 (14)	C31—P2—C25	108.67 (16)
C1—P1—C4	107.97 (15)	C22—P2—C25	107.82 (16)
C1—P1—C16	111.24 (16)	C22—P2—C37	111.71 (17)
C1—P1—C10	111.25 (15)	C22—P2—C31	109.93 (16)
C79—B1—C73	109.3 (2)	C26—C25—P2	118.5 (3)
C79—B1—C67	107.4 (2)	C26—C25—C30	120.9 (3)
C67—B1—C73	111.3 (3)	C30—C25—P2	120.7 (3)
C85—B1—C79	111.1 (3)	C42—C37—P2	120.9 (3)
C85—B1—C73	106.7 (3)	C42—C37—C38	119.8 (3)
C85—B1—C67	111.0 (2)	C38—C37—P2	119.2 (3)
C67—C72—H72	118.8	C36—C31—P2	120.0 (3)
C71—C72—H72	118.8	C32—C31—P2	119.7 (3)
C71—C72—C67	122.4 (3)	C32—C31—C36	120.2 (3)
C18—C17—H17	120.0	C36—C35—H35	119.5
C18—C17—C16	120.0 (3)	C34—C35—H35	119.5
C16—C17—H17	120.0	C34—C35—C36	121.0 (3)
C80—C79—B1	125.5 (3)	C31—C36—H36	120.7
C80—C79—C84	114.3 (3)	C35—C36—C31	118.7 (3)
C84—C79—B1	120.3 (3)	C35—C36—H36	120.7
C1—C2—H2	117.1	C25—C26—H26	120.5
C1—C2—C3	125.7 (3)	C25—C26—C27	119.0 (4)
C3—C2—H2	117.1	C27—C26—H26	120.5
C5—C4—P1	118.3 (3)	C31—C32—H32	120.2
C9—C4—P1	122.1 (3)	C31—C32—C33	119.5 (3)
C9—C4—C5	119.6 (3)	C33—C32—H32	120.2
C17—C18—H18	119.7	C22—C23—H23	117.2
C17—C18—C19	120.5 (3)	C22—C23—C24	125.5 (3)
C19—C18—H18	119.7	C24—C23—H23	117.2
C74—C73—B1	126.3 (3)	C37—C42—H42	119.9
C74—C73—C78	115.0 (3)	C41—C42—C37	120.2 (3)
C78—C73—B1	118.6 (3)	C41—C42—H42	119.9
C72—C67—B1	125.4 (3)	C35—C34—H34	120.1
C72—C67—C68	114.6 (3)	C33—C34—C35	119.7 (3)
C68—C67—B1	120.0 (3)	C33—C34—H34	120.1
C16—C21—H21	119.4	C42—C41—H41	120.3
C20—C21—H21	119.4	C40—C41—C42	119.4 (4)
C20—C21—C16	121.3 (3)	C40—C41—H41	120.3
C17—C16—P1	119.2 (2)	C37—C38—H38	120.2
C21—C16—P1	122.2 (2)	C39—C38—C37	119.7 (4)
C21—C16—C17	118.6 (3)	C39—C38—H38	120.2
C10—C11—H11	120.6	C30—C29—H29	120.1
C12—C11—H11	120.6	C28—C29—H29	120.1
C12—C11—C10	118.8 (3)	C28—C29—C30	119.7 (4)
C11—C10—P1	120.6 (3)	C41—C40—H40	119.5
C15—C10—P1	119.5 (2)	C41—C40—C39	120.9 (4)
C15—C10—C11	119.9 (3)	C39—C40—H40	119.5
C4—C5—H5	119.9	P2—C22—H22	117.2
C6—C5—C4	120.2 (3)	C23—C22—P2	125.6 (3)
C6—C5—H5	119.9	C23—C22—H22	117.2
C74—C75—H75	119.8	C23—C24—H24A	109.5
C76—C75—H75	119.8	C23—C24—H24B	109.5
C76—C75—C74	120.4 (3)	C23—C24—H24C	109.5
C4—C9—H9	120.3	H24A—C24—H24B	109.5
C4—C9—C8	119.4 (3)	H24A—C24—H24C	109.5
C8—C9—H9	120.3	H24B—C24—H24C	109.5
C67—C68—H68	118.2	C25—C30—C29	119.2 (4)
C69—C68—C67	123.6 (3)	C25—C30—H30	120.4
C69—C68—H68	118.2	C29—C30—H30	120.4
C79—C80—H80	118.5	C32—C33—H33	119.6
C81—C80—C79	123.0 (3)	C34—C33—C32	120.8 (4)
C81—C80—H80	118.5	C34—C33—H33	119.6
C88—C87—H87	119.4	C29—C28—H28	119.8
C88—C87—C86	121.2 (4)	C27—C28—C29	120.4 (4)
C86—C87—H87	119.4	C27—C28—H28	119.8
C13—C14—H14	119.7	C38—C39—C40	119.9 (4)
C13—C14—C15	120.5 (4)	C38—C39—H39	120.0
C15—C14—H14	119.7	C40—C39—H39	120.0
P1—C1—H1	117.2	C26—C27—H27	119.6
C2—C1—P1	125.6 (3)	C28—C27—C26	120.8 (4)
C2—C1—H1	117.2	C28—C27—H27	119.6
C84—C83—H83	119.8	C66—C61—B2	123.5 (3)
C82—C83—H83	119.8	C62—C61—C66	114.7 (3)
C82—C83—C84	120.4 (3)	C62—C61—B2	121.6 (3)
C69—C70—H70	120.6	C51—C52—H52	120.6
C69—C70—C71	118.8 (3)	C51—C52—C53	118.8 (3)
C71—C70—H70	120.6	C53—C52—H52	120.6
C79—C84—H84	118.5	C49—C54—H54	118.5
C83—C84—C79	123.0 (3)	C53—C54—H54	118.5
C83—C84—H84	118.5	C53—C54—C49	122.9 (3)
C78—C77—H77	119.9	C61—C66—H66	118.8
C76—C77—H77	119.9	C65—C66—C61	122.4 (3)
C76—C77—C78	120.1 (3)	C65—C66—H66	118.8
C73—C74—C75	122.8 (3)	C51—C50—H50	118.2
C73—C74—H74	118.6	C51—C50—C49	123.6 (3)
C75—C74—H74	118.6	C49—C50—H50	118.2
C5—C6—H6	119.9	C48—C43—B2	121.9 (3)
C7—C6—C5	120.1 (3)	C44—C43—C48	114.0 (3)
C7—C6—H6	119.9	C44—C43—B2	124.1 (3)
C90—C85—B1	118.3 (3)	C52—C51—C50	120.3 (3)
C86—C85—B1	126.5 (3)	C52—C51—H51	119.9
C86—C85—C90	115.2 (3)	C50—C51—H51	119.9
C2—C3—H3A	109.5	C56—C55—C60	113.2 (3)
C2—C3—H3B	109.5	C56—C55—B2	125.2 (3)
C2—C3—H3C	109.5	C60—C55—B2	121.6 (3)
H3A—C3—H3B	109.5	C54—C49—B2	120.4 (3)
H3A—C3—H3C	109.5	C50—C49—C54	114.4 (3)
H3B—C3—H3C	109.5	C50—C49—B2	125.1 (3)
C80—C81—H81	119.7	C55—C56—H56	118.1
C82—C81—C80	120.5 (3)	C55—C56—C57	123.8 (4)
C82—C81—H81	119.7	C57—C56—H56	118.1
C18—C19—H19	120.1	C62—C63—H63	120.4
C20—C19—C18	119.7 (3)	C64—C63—H63	120.4
C20—C19—H19	120.1	C64—C63—C62	119.2 (3)
C14—C13—H13	120.0	C61—C62—C63	123.7 (3)
C12—C13—C14	119.9 (4)	C61—C62—H62	118.1
C12—C13—H13	120.0	C63—C62—H62	118.1
C68—C69—H69	120.2	C55—C60—H60	117.9
C70—C69—C68	119.6 (3)	C59—C60—C55	124.2 (4)
C70—C69—H69	120.2	C59—C60—H60	117.9
C85—C90—H90	118.5	C52—C53—C54	120.0 (3)
C89—C90—C85	123.0 (3)	C52—C53—H53	120.0
C89—C90—H90	118.5	C54—C53—H53	120.0
C83—C82—C81	118.8 (3)	C43—C48—H48	118.6
C83—C82—H82	120.6	C47—C48—C43	122.8 (4)
C81—C82—H82	120.6	C47—C48—H48	118.6
C10—C15—H15	120.2	C43—C44—H44	118.3
C14—C15—C10	119.7 (3)	C45—C44—C43	123.3 (4)
C14—C15—H15	120.2	C45—C44—H44	118.3
C73—C78—H78	118.7	C60—C59—H59	119.9
C77—C78—C73	122.6 (3)	C58—C59—C60	120.1 (4)
C77—C78—H78	118.7	C58—C59—H59	119.9
C9—C8—H8	119.6	C56—C57—H57	120.0
C7—C8—C9	120.7 (3)	C58—C57—C56	120.1 (4)
C7—C8—H8	119.6	C58—C57—H57	120.0
C87—C88—H88	120.7	C59—C58—H58	120.7
C87—C88—C89	118.7 (4)	C57—C58—C59	118.5 (4)
C89—C88—H88	120.7	C57—C58—H58	120.7
C90—C89—C88	119.5 (3)	C66—C65—H65	119.7
C90—C89—H89	120.3	C64—C65—C66	120.6 (3)
C88—C89—H89	120.3	C64—C65—H65	119.7
C21—C20—H20	120.1	C61—B2—C43	107.8 (3)
C19—C20—C21	119.9 (3)	C61—B2—C55	111.1 (3)
C19—C20—H20	120.1	C61—B2—C49	108.6 (3)
C6—C7—C8	119.9 (3)	C55—B2—C43	108.9 (3)
C6—C7—H7	120.1	C49—B2—C43	110.4 (3)
C8—C7—H7	120.1	C49—B2—C55	110.1 (3)
C87—C86—H86	118.8	C63—C64—H64	120.3
C85—C86—C87	122.5 (3)	C65—C64—C63	119.3 (3)
C85—C86—H86	118.8	C65—C64—H64	120.3
C75—C76—C77	119.0 (3)	C45—C46—H46	120.7
C75—C76—H76	120.5	C47—C46—H46	120.7
C77—C76—H76	120.5	C47—C46—C45	118.7 (4)
C72—C71—H71	119.5	C44—C45—H45	119.8
C70—C71—C72	121.0 (3)	C46—C45—C44	120.4 (4)
C70—C71—H71	119.5	C46—C45—H45	119.8
C11—C12—H12	119.4	C48—C47—H47	119.6
C13—C12—C11	121.2 (4)	C46—C47—C48	120.7 (4)
C13—C12—H12	119.4	C46—C47—H47	119.6
