# Supplementary figures and images for: Crystal structure of tri­phenyl(vinyl)­phospho­nium tetra­phenyl­borate
Source: Acta Crystallogr Sect E Struct Rep Online. 2014 Sep 30;70(Pt 10):o1143. doi: 10.1107/S1600536814021357 (PMC4257183; doi:10.1107/S1600536814021357)

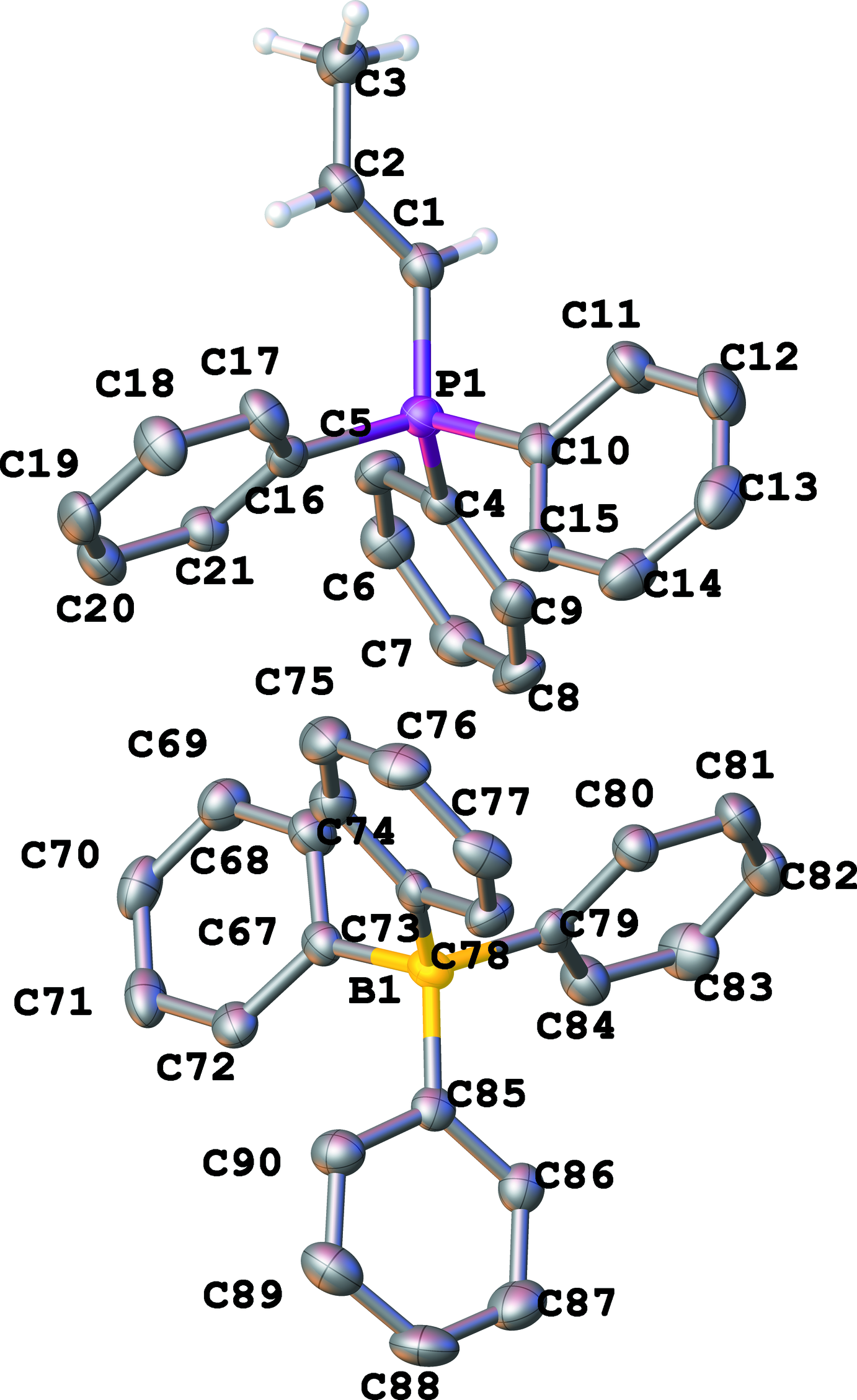

Supplement: Supplementary file 3 [file e-70-o1143-fig1.tif]

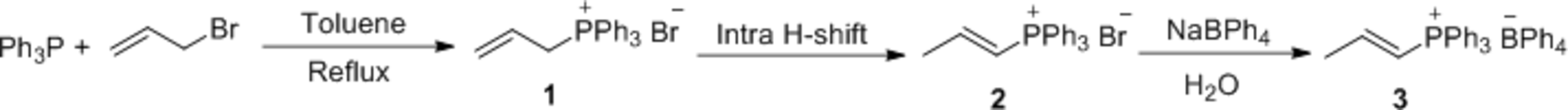

Supplement: Supplementary file 4 [file e-70-o1143-fig2.tif]
